# Association of follicle stimulating hormone and serum lipid profiles in postmenopausal women

**DOI:** 10.1097/MD.0000000000030920

**Published:** 2022-09-30

**Authors:** Zhengfen Xu, Shuiqin Gu, Xiaojie Wu, Ying Zhou, Huan Li, Xuedong Tang

**Affiliations:** a Department of Obstetrics and Gynecology, Jiaxing Maternity and Child Health Care Hospital, College of Medicine, Jiaxing University, Jiaxing, China; b Department of Nursing, Jiaxing Maternity and Child Health Care Hospital, College of Medicine, Jiaxing University, Jiaxing, China.

**Keywords:** cardiovascular disease, follicle stimulating hormone, lipid profiles, postmenopausal women

## Abstract

The aim of the study was to observe the association between follicle stimulating hormone (FSH) levels and serum lipid profiles in postmenopausal women. A total of 411 healthy postmenopausal women with a mean age of 55 years (range 45–65 years) were enrolled in this study. Data on age, time of last menstrual period, past medical history, use of medications, and smoking status were collected, and body weight, height, and blood pressure were measured. Blood samples were collected to measure the serum concentrations of FSH, luteinizing hormone (LH), estradiol (E2), glucose, total cholesterol (TC), triglyceride (TG), high-density lipoprotein cholesterol (HDL-C), and low-density lipoprotein cholesterol (LDL-C) using routine methods. FSH levels were negatively associated with LDL-C, even after adjustment for age, LH, E2, BMI, systolic blood pressure (SBP), and diastolic blood pressure (DBP) (OR = 0.185, 95% CI = 0.051‐0.669). Although FSH may also be negatively associated with dyslipidemia (*P* = .06 for trend) and hypercholesterolemia (*P* = .079 for trend), but no statistical significance was found after adjusting for confounding factors, particularly BMI. All relevant data are within the paper and its Supporting Information files. The results indicated that lower FSH levels might increase the odds of dyslipidemia, especially the risk of LDL-C elevation, which is an important factor that increases the risk of CVD in postmenopausal women.

## 1. Introduction

Cardiovascular disease (CVD) threatens women’s health and is the leading cause of death in postmenopausal women. Previous studies have showed that dyslipidemia is one of the most important risk factors of CVD, and after menopause, the incidence of dyslipidemia increases sharply.^[1]^ Dyslipidemia in menopause is characterized by an increase in low-density lipoprotein (LDL) levels and a decline in high-density lipoprotein (HDL) levels.^[2]^ These changes have a clear negative impact on the cardiovascular system, accelerating the development of CVD.^[2]^

Previous studies focused on the relationship between dyslipidemia and estrogen deficiency in postmenopausal women, but latest studies have found that endogenous estrogens were not independent predictors.^[3]^ We all know that the level of estrogen is generally low in postmenopausal women, but the level of follicle stimulating hormone (FSH) is variable, especially in obese and lean women. Some studies found that hormone replace therapy (HRT) had no significant impact on lipid profiles.^[4]^ Recent studies focus on the association between FSH and cardiometabolic factors including serum lipids, but the results are controversial whether FSH is negatively or positively associated with dyslipidemia in postmenopausal women.

So, we conducted the cross-sectional study to further observe whether changes in FSH were related to dyslipidemia in postmenopausal women. Hopefully, this will be helpful for the management of cardiovascular risk and dyslipidemias in postmenopausal women.

## 2. Materials and Methods

### 2.1. Subjects

A total of 411 healthy menopausal women between the ages of 45 and 65 who went to the Health Examination Center at Jiaxing Maternity and Child Health Care Hospital from January 2019 to December 2020 were enrolled in the cross-sectional study. Women with amenorrhea for at least 12 months were included in the study. The exclusion criteria was as follows: FSH < 25 IU/L, abnormal uterus bleeding, artificial menopause, use of hormones or drugs that affect menopausal status and lipid levels within past 3 months and smoking. This study was approved by the ethics committee of Jiaxing Maternity and Child Health Care Hospital. All participants signed an informed consent form.

### 2.2. Data collection

The basic information including age, time of last menstrual period, past medical history, use of medications was gathered by one training doctor. Body weight, height, and blood pressure were measured using standard protocols by one training nurse. Blood samples were obtained in the morning after at least 12 hours of fasting and were tested in the laboratory of our hospital that day. Serum concentrations of FSH, luteinizing hormone (LH) and estradiol (E2) were measured using chemiluminescent immunoassays (Abbott, USA); Serum lipids concentrations were measured using an enzymatic colorimetric assay (Abbott, USA).

### 2.3. Definition of variables

According to the Adult Treatment Panel (ATP) III guidelines,^[5]^ total cholesterol (TC) ≥ 6.20 mmol/L was defined as abnormal TC; Triglyceride ≥ 2.3 mmol/L was defined as abnormal triglyceride (TG); low-density lipoprotein cholesterol ≥ 4.1 mmol/L was defined as abnormal LDL-C; high-density lipoprotein cholesterol < 1.0 mmol/L was defined as abnormal HDL-C; dyslipidemia was defined as any abnormality of one or more of the four serum lipid concentrations above.

### 2.4. Statistical analysis

Data was analyzed in IBM SPSS Statistics 25 (IBM, New York). Normal distribution of data was checked using Shapiro–Wilk test. Continuous variables conforming to a normal distribution were reported as mean ± standard deviation and compared between groups by analysis of variance. Otherwise, median (interquartile range) and the nonparametric test were used. Categorical variables were reported as numbers and proportion (%). Linear regression was used to evaluate the association between FSH and lipids levels and 4 models were built to adjust for potential confounders including age and BMI. We then used multivariate logistic regression to analyze the association of FSH levels and dyslipidemia in fully adjusted models. *P* < .05 was considered statistically significant.

## 3. Results

### 3.1. Characteristics according to FSH quartiles in postmenopausal women

A total of 411 women participated in the study. The average age was 56.3 ± 4.5 years. The average age at menopause was 50.0 ± 3.6 years and the average menopausal period was 6.3 ± 4.7 years. The estrogen level of postmenopausal women was relatively low and stable, so we chose them as participants to reduce the impact of estrogen on the study. Participants were divided into four groups according to quartiles of FSH levels. The estrogen levels were very low in all four groups and showed no significant differences. With the increase of FSH quartiles, the levels of BMI, LH, TC, TG and LDL-C decreased gradually and the level of HDL-C increased gradually, the difference was statistically significant (*P* < .001). There were also significant differences in ages, menopausal years, systolic and diastolic blood pressure (DBP) between the four groups (*P* < .05) (Table 1).

**Table 1:** Characteristics according to FSH quartiles in postmenopausal women.

FSH quartile (n)	Quartile 1 (n = 103)	Quartile 2 (n = 103)	Quartile 3 (n = 103)	Quartile 4 (n = 102)	*P*
FSH range (IU/L)	≤43.22	43.23‐54.53	54.54‐68.44	≥68.45	
Age (yr)	57.46 ± 4.54	56.48 ± 4.52	56.24 ± 3.98	55.07 ± 4.72^ab^	.002
Menopause age (yr)	50 (48‐52)	51 (48‐53)	50.00 (48‐52)	50.00 (48‐52)	.655
Menopausal years (yr)	7 (2.5‐12)	5 (3‐8.5)	5 (2‐9.5)	4 (2‐7)^a^	.023
BMI (kg/m^2^)	25.06 ± 3.09	23.54 ± 2.88^a^	22.59 ± 2.53^ab^	22.20 ± 2.49^ab^	<.001
SBP (mm Hg)	137.20 ± 15.47	130.77 ± 16.95^a^	129.77 ± 15.12^a^	129.16 ± 16.29^a^	.001
DBP (mm Hg)	85.37 ± 9.19	81.15 ± 9.82^a^	81.50 ± 8.58^a^	80.63 ± 10.28^a^	.001
E2 (pg/mL)	6.37 (5.66‐8.27)	6.11 (5.36‐7.14)	5.98 (5.31‐6.56)	5.78 (5.18‐6.77)	.085
LH (IU/L)	14.12 (11.81‐17.96)	19.49 (15.98‐23.05)^a^	23.58 (19.55‐27.32)^ab^	31.17 (25.58‐37.91)^abc^	<.001
TC (mmol/L)	6.16 (4.51‐6.93)	4.64 (4.17‐5.99)^a^	4.62 (3.96‐5.49)^a^	4.62 (4.23‐5.23)^a^	<.001
TG (mmol/L)	2.31 (1.37‐2.65)	1.60 (0.99‐2.30)^a^	1.38 (1.05‐2.07)^a^	1.41 (1.00‐2.24)^a^	<.001
HDL-C (mmol/L)	1.19 (1.03‐1.37)	1.28 (1.10‐1.50)	1.30 (1.07-1.48)	1.40 (1.13‐1.59)^a^	<.001
LDL-C (mmol/L)	3.98 (2.77-4.46)	2.86 (2.32‐4.16)^a^	2.79 (2.39‐3.44)^a^	2.74 (2.31‐3.34)^a^	<.001
Dyslipidemia, N (%)	69 (67.0%)	42 (40.8%)	39 (37.9%)	30 (29.4%)	<.001

The data are expressed as mean ± standard deviation, median (interquartile range), number (percentage) and *P* value was calculated by ANOVA and *χ*^2^ test.

BMI = body mass index; DBP = diastolic blood pressure; E2 = estradiol; FSH = follicle stimulating hormone; HDL = high-density lipoprotein; LDL = low-density lipoprotein; LH = luteinizing hormone; SBP = systolic blood pressure; TC = total cholesterol; TG = triglycerides.

a Compared with FSH quartile1 (*P* < .05).

b Compared with FSH quartile2 (*P* < .05).

c Compared with FSH quartile3 (*P* < .05).

### 3.2. Association of FSH and lipids levels in postmenopausal women

In unadjusted linear regression models, FSH levels were negatively associated with TC, TG and LDL-C, and positively associated with HDL-C. After adjusting for age, LH and E2, the association with TC was attenuated but remained statistically significant, yet the association with TG and HDL-C was no longer significant. In fully adjusted models, the association with LDL-C was attenuated but still statistically significant, however the association with TC was no longer significant (Table 2).

**Table 2:** Association of FSH and lipids levels in postmenopausal women.

	B	SE	95%CI of B	Beta	*P*
TC					
unadjusted	–0.017	0.003	–0.023 to 0.011	–0.264	<.001
Model 1	–0.017	0.003	–0.023 to 0.011	–0.267	<.001
Model 2	–0.016	0.005	–0.025 to –0.007	–0.250	<.001
Model 3	–0.006	0.004	–0.014 to 0.002	–0.089	.164
TG					
unadjusted	–0.010	0.003	–0.016 to –0.004	–0.163	.001
Model 1	–0.012	0.003	–0.018 to –0.005	–0.184	<.001
Model 2	–0.009	0.005	–0.018 to 0.000	–0.138	.058
Model 3	–0.003	0.005	–0.012 to 0.006	–0.045	.535
HDL-C					
unadjusted	0.003	0.001	0.002 to 0.005	0.201	<.001
Model 1	0.003	0.001	0.002 to 0.005	0.197	<.001
Model 2	0.003	0.001	0.000 to 0.005	0.163	.025
Model 3	0.002	0.001	–0.001 to 0.004	0.102	.162
LDL-C					
unadjusted	–0.013	0.002	–0.018 to –0.008	–0.264	<.001
Model 1	–0.013	0.002	–0.018 to –0.009	–0.272	<.001
Model 2	–0.014	0.003	–0.021 to –0.007	–0.290	<.001
Model 3	–0.007	0.003	–0.013 to –0.001	-0.139	.034

Data are expressed as unstandardized coefficients (B), corresponding standard error (SE), 95% confidence interval (CI) of B, standardized coefficients (Beta), and significance (*P* value). Model 1 adjusted for age; Model 2 adjusted for age, LH, E2; Model 3 adjusted for age, LH, E2, BMI, SBP, DBP.

BMI = body mass index, DBP = diastolic blood pressure, E2 = estradiol, FSH = follicle stimulating hormone, HDL = high-density lipoprotein, LH = luteinizing hormone, LDL = low-density lipoprotein, TC = total cholesterol, TG = triglycerides, SBP = systolic blood pressure.

### 3.3. Association of FSH levels with the risk of abnormal lipids levels in postmenopausal women

**Figure 1:**
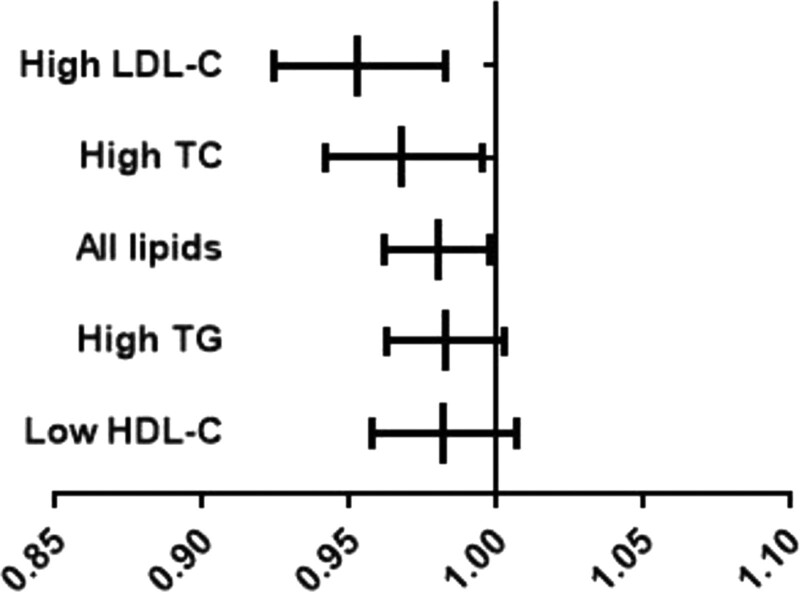
Association of FSH levels with the risk of abnormal lipids levels in postmenopausal women. Adjusted by age, BMI, LH, E2, SBP, DBP. BMI = body mass index, DBP = diastolic blood pressure, E2 = estradiol, FSH = follicle stimulating hormone, HDL = high-density lipoprotein, LH = luteinizing hormone, LDL = low-density lipoprotein, TC = total cholesterol, TG = triglycerides, SBP = systolic blood pressure.

A binary logistic regression model was used to analyze the association of FSH levels and the risk of dyslipidemia and any abnormality of lipid components. The result indicated that higher levels of FSH had a significantly protective effect on LDL-C (95% CI 0.924‐0.983, *P* = .002) and slight protective effects on all lipids (95% CI 0.962‐0.998, *P* = .03) and TC (95% CI 0.942‐0.995, *P* = .019) after adjusting for age, BMI, LH, E2, SBD, DBD. We didn’t find significant associations between FSH levels with HDL-C and TG (Table 3, Fig. 1).

**Table 3:** Association of FSH levels with the risk of abnormal lipids levels in postmenopausal women.

	N (%)	B	SE	OR (95%CI)	*P*
All lipids					
Normal	231 (56.2%)				
Abnormal	180 (43.8%)	–0.020	0.009	0.980 (0.962–0.998)	.030
TC					
Normal	309 (75.18%)				
Abnormal	102 (24.82%)	–0.032	0.014	0.968 (0.942‐0.995)	.019
TG					
Normal	289 (70.32%)				
Abnormal	122 (29.68%)	–0.018	0.010	0.983 (0.963‐1.003)	.089
HDL-C					
Normal	351 (85.4%)				
Abnormal	60 (14.6%)	–0.018	0.013	0.982 (0.958‐1.007)	.150
LDL-C					
Normal	338 (82.24%)				
Abnormal	73 (17.76%)	–0.048	0.016	0.953 (0.924‐0.983)	.002

Data are expressed as number (percentage), unstandardized coefficients (B), corresponding standard error (SE), adjusted odds ratio (OR), 95% confidence interval (CI), significance (*P* value). Lipid classifications based on ATP III guidelines. Adjusted by age, BMI, LH, E2, SBP, DBP.

BMI = body mass index, DBP = diastolic blood pressure, E2 = estradiol, FSH = follicle stimulating hormone, HDL = high-density lipoprotein, LH = luteinizing hormone, LDL = low-density lipoprotein, TC = total cholesterol, TG = triglycerides, SBP = systolic blood pressure.

### 3.4. Association of FSH quartiles with the risk of abnormal lipids levels in postmenopausal women

For further observation the association between FSH and serum lipids, we performed a multivariate logistic regression with full adjustment. Comparing the highest with the lowest FSH quartile, a risk reduction of 81.5% was shown for LDL-C anomaly (OR = 0.185, 95%CI = 0.051‐0.669). We also observed a slight but not statistically significant decrease in all lipids (*P* = .06 for trend) and TC (*P* = .079 for trend) compared with those in the lowest FSH quartile. No statistically significant trend association was found between FSH quartiles with TG (*P* for trend = 0.189) and HDL-C (*P* for trend = 0.117) (Table 4).

**Table 4:** Association of FSH quartiles with the risk of abnormal lipids levels in postmenopausal women.

	B	SE	OR (95%CI)	*P*	*p* for trend
All lipids					.061
FSH Q1					
FSH Q2	–0.784	0.331	0.457 (0.239‐0.873)	.018	
FSH Q3	–0.618	0.357	0.539 (0.268‐1.084)	.083	
FSH Q4	–0.934	0.439	0.393 (0.166‐0.929)	.033	
Total cholesterol					.079
FSH Q1					
FSH Q2	–0.846	0.401	0.429 (0.195‐0.942)	.035	
FSH Q3	–0.632	0.447	0.532 (0.221‐1.278)	.158	
FSH Q4	–1.053	0.571	0.349 (0.114‐1.069)	.065	
TG					.189
FSH Q1					
FSH Q2	–0.795	0.338	0.451 (0.233‐0.876)	.019	
FSH Q3	–0.788	0.377	0.455 (0.217‐0.953)	.037	
FSH Q4	–0.514	0.455	0.598 (0.245‐1.459)	.259	
HDL-C					.117
FSH Q1					
FSH Q2	–1.240	0.447	0.290 (0.120‐0.696)	.006	
FSH Q3	–0.427	0.428	0.652 (0.282‐1.510)	.319	
FSH Q4	–1.245	0.603	0.288 (0.088‐0.938)	.039	
LDL-C					.010
FSH Q1					
FSH Q2	–1.084	0.430	0.338 (0.146‐0.785)	.012	
FSH Q3	–0.912	0.482	0.402 (0.156‐1.033)	.058	
FSH Q4	–1.685	0.655	0.185 (0.051‐0.669)	.010	

Data are expressed as unstandardized coefficients (B), corresponding standard error (SE), adjusted odds ratio (OR), 95% confidence interval (CI) of B and significance (*P* value and *p* for trend). Adjusted by age, BMI, LH, E2, SBP, DBP.

BMI = body mass index, DBP = diastolic blood pressure, E2 = estradiol, FSH = follicle stimulating hormone, HDL = high-density lipoprotein, LH = luteinizing hormone, LDL = low-density lipoprotein, TC = total cholesterol, TG = triglycerides, SBP = systolic blood pressure.

## 4. Discussion

The goal of the study was to find the association between FSH levels and serum lipid profiles in postmenopausal women. We found that FSH levels were negatively associated with LDL-C, even after adjustment for age, LH, E2, BMI, systolic blood pressure (SBP), and DBP. Although FSH may also be negatively associated with dyslipidemia and hypercholesterolemia, but no statistical significance was found after adjusting for confounding factors, particularly BMI. These findings suggest that lower FSH levels may increase the odds of dyslipidemia, especially the risk of LDL-C elevation, which is an important factor that increases the risk of CVD.

Following menopause, women exhibit an increased prevalence of dyslipidemia, especially high levels of LDL-C and TC, which can lead to CVD.^[6]^ Previous studies have focused on the relationship between dyslipidemia and estrogen deficiency in postmenopausal women; however, recent studies have found that endogenous estrogens are not independent predictors of lipid levels in postmenopausal women.^[3]^ In our study, the estrogen levels were not statistically different among the different FSH quartiles. Some studies found that HRT had no significant impact on the lipid profile, and some women still developed dyslipidemia despite HRT.^[4]^ Other findings from large randomized trials also do not confirm the benefit of estrogen therapy for the prevention of cardiovascular disease, and HRT is not recommended for this purpose in clinical practice.^[7]^ Clinically, we also found that by using HRT, although FSH decreased, no effect on the lipid profile was found. This phenomenon may also indicate that low FSH levels may be a risk factor for dyslipidemia, which is consistent with previous clinical investigations. To determine whether low FSH levels had an independent association with serum lipid profiles, we classified postmenopausal women into FSH quartiles. We found that as FSH levels decreased, the levels of TC, TG, and LDL-C gradually increased, and HDL-C levels gradually decreased; the difference was statistically significant. The estrogen levels of the women were extremely low and showed no significant differences among the FSH quartiles. Therefore, our findings provide new evidence for the role of FSH in metabolic disorders in postmenopausal women.

In this study, BMI also increased gradually as FSH levels decreased, and this difference was statistically significant. Previous studies have found that obesity had an inhibitory effect on FSH levels^[8]^ and weight loss decreased FSH levels in overweight postmenopausal women.^[9]^ These findings may exist because there may be due to a possible inhibitory effect of body mass on gonadotropin.^[10]^ However, in our study, the association between FSH and LDL-C remained significant even after adjusting for BMI, indicating that the association between FSH and LDL-C was independent of BMI. Studies have suggested that obesity, as a pandemic in the modern world, is intimately associated with dyslipidemia.^[11]^ This could explain the attenuation associations between FSH and TC, TG, and HDL-C levels after adjusting for BMI. However, further investigation is required to confirm this hypothesis.

Other studies have supported the opinion that low FSH levels may be a risk factor for dyslipidemia.^[12–14]^ A study including 2658 Chinese postmenopausal women reported a direct association between FSH and HDL-C and an inverse relationship between TG and LDL-C.^[15]^ Another study did not find decrements in serum cholesterol after eight weeks of anti-FSH antibody treatment.^[16]^ To date, only a few studies have investigated the relationship between FSH levels and serum lipid profiles, and the results have been controversial. Several studies have reported diametrically opposite effects of FSH on dyslipidemia.^[17–19]^ To further substantiate the role of FSH in cholesterol metabolism, Guo et al used ovariectomized mice, in which estrogen was clamped by exogenous administration. They found that when injected with recombinant FSH, these mice showed higher levels of serum TC and LDL-C, and elevated hepatic cholesterol biosynthesis.^[17]^

The INTERHEART study, which enrolled 52 countries worldwide, including China, showed that abnormal lipid levels are important risk factors for cardiovascular disease.^[6]^ An assessment based on the Systematic Coronary Risk Estimation (SCORE) system of the 10-year risk of fatal cardiovascular disease suggested that LDL-C should be the optimal target in clinical practice.^[20]^ LDL contributes to the formation of plaques, which are thick, hard deposits that can clog the arteries, thereby compromising the flexibility of arteries and resulting in atherosclerosis.^[21]^ A previous study suggested that a reduction of LDL cholesterol by 2 to 3 mmol/L would reduce CVD risk by approximately 40% to 50%.^[22]^ In our study, after adjustment for full confounders, the association between FSH and LDL-C remained significantly the same as that in the unadjusted models. These findings suggest that the levels of FSH and LDL-C should be emphasized during HRT. We should use the lowest effective dose of estrogen, which could alleviate menopausal syndrome and not lower FSH to very low levels.

This study has some limitations. First, our study was only a cross-sectional observational study with a limited number of participants; second, we did not observe an association between FSH and other lipoproteins, such as apolipoprotein B and lipoprotein (a); third, the age span was not large enough and women with POI were not included.

In summary, to promote dyslipidemia management and effectively reduce the risk of CVD in postmenopausal women, studies involving different geographic regions, ethnic groups, and various lipid profiles should be conducted to determine the exact relationship and mechanism of action of FSH with serum lipids.

## 5. Conclusions

FSH may be associated with serum lipids in postmenopausal women; therefore, attention should be paid to FSH and LDL-C levels when HRT is administered.

## Acknowledgments

We are very thankful to Dr Pengfei Shan for reviewing this manuscript.

## Authors contribution

**Conceptualization:** Zhengfen Xu.

**Data curation:** Shuiqin Gu, Ying Zhou.

**Investigation:** Xiaojie Wu, Huan Li.

**Methodology:** Shuiqin Gu.

**Resources:** Ying Zhou.

**Software:** Ying Zhou.

**Supervision:** Xuedong Tang.

**Writing – original draft:** Zhengfen Xu.

**Writing – review &amp; editing:** Xuedong Tang.
